# COVID‐19 and coagulation dysfunction in adults: A systematic review and meta‐analysis

**DOI:** 10.1002/jmv.26346

**Published:** 2020-08-02

**Authors:** Jing Lin, Han Yan, Hanchuan Chen, Chen He, Chunjin Lin, Haoming He, Sicheng Zhang, Songjing Shi, Kaiyang Lin

**Affiliations:** ^1^ Department of Critical Care Medicine, Fujian Provincial Hospital, Shengli Clinical Medical College Fujian Medical University Fuzhou China; ^2^ Department of Cardiology, Fujian Provincial Hospital, Shengli Clinical Medical College Fujian Medical University Fuzhou China

**Keywords:** 2019 novel coronavirus disease, coagulation, meta‐analysis, severity

## Abstract

The outbreak of 2019 novel coronavirus disease (COVID‐19) has posed a grave threat to the global public health. The COVID‐19‐induced infection is closely related to coagulation dysfunction in the affected patients. This paper attempts to conduct a meta‐analysis and systematically review the blood coagulation indicators in patients with severe COVID‐19. A meta‐analysis of eligible studies was performed to compare the blood coagulation indicators in patients with severe and nonsevere COVID‐19. PubMed, Embase, Web of Science, and the Cochrane Library were searched for studies published between 1 December 2019 and 7 May 2020. A total of 13 studies with 1341 adult patients were enrolled in this analysis. Platelet (weighted mean difference [WMD] = −24.83, 95% confidence interval [CI]: −34.12 to −15.54; *P* < .001), d‐dimer (WMD = 0.19, 95% CI: 0.09‐0.29; *P* < .001), and fibrinogen (WMD = 1.02, 95% CI: 0.50‐1.54; *P* < .001) were significantly associated with the severity in patients with COVID‐19. The meta‐analysis revealed that no correlation was evident between an increased severity risk of COVID‐19 and activated partial thromboplastin time (WMD = −1.56, 95% CI: −5.77 to 2.64; *P* = .468) or prothrombin time (WMD = 0.19, 95% CI: −0.13 to 0.51; *P* = .243). The single arm meta‐analysis showed that compared with the nonsevere group, the severe group had a lower pooled platelet (165.12 [95% CI: 157.38‐172.85] vs 190.09 [95% CI: 179.45‐200.74]), higher d‐dimer (0.49 [95% CI: 0.33‐0.64] vs 0.27 [95% CI: 0.20‐0.34]), and higher fibrinogen (4.34 [95% CI: 1.98‐6.70] vs 3.19 [95% CI: 1.13‐5.24]). Coagulation dysfunction is closely related to the severity of patients with COVID‐19, in which low platelet, high d‐dimer, and fibrinogen upon admission may serve as risk indicators for increased aggression of the disease. These findings are of great clinical value for timely and effective treatment of the COVID‐19 cases.

## Highlights

- Coagulation dysfunction affects the prognosis of COVID‐19 patients.
- Lower platelet, higher d‐dimer and fibrinogen indicate increased severity risk in COVID‐19 patients.
- No difference in PT and APTT is evident between severe and non‐severe COVID‐19 patients upon admission.

**Abbreviations**
APTT: activated partial thromboplastin time
ARDS: acute respiratory distress syndrome
ATS: American Thoracic Society
COVID‐19: 2019 novel coronavirus disease
DIC: disseminated intravascular coagulation
ICU: intensive care unit
NOS: Newcastle‐Ottawa Scale
PE: pulmonary thromboembolism
PT: prothrombin time
RR: respiration rate
SARS: severe acute respiratory syndrome
SARS‐CoV‐2: severe acute respiratory syndrome coronavirus 2
SD: standard deviation
VTE: venous thromboembolism
WMD: weighted mean difference
95% CI: 95% confidence interval

## 1 INTRODUCTION

The global outbreak of 2019 novel coronavirus disease (COVID‐19) has posed enormous impacts on the public health, with severe and critically ill patients accounting for 20% of all COVID‐19 patients^1^ and the fatality rate of critically ill patients amounting to 49%.^2^ Severe patients are more likely to develop acute respiratory distress syndrome (ARDS) and multiple organ dysfunction syndrome, which may affect the prognosis of patients with COVID‐19.^3^ Therefore, an early screening of severe and nonsevere patients is critical to reduce the mortality rate of patients with COVID‐19.

Of note, abnormal coagulation function has been prevalent in 20% of the patients with COVID‐19.^4^ Moreover, the prevalence of disseminated intravascular coagulation (DIC) in COVID‐19 cases is higher than that of severe acute respiratory syndrome (SARS) patients.^5^ A recent study reports that the mortality of COVID‐19‐induced DIC is 71.4%.^6^ Hence, coagulation dysfunction is closely related to the severity of COVID‐19 cases and can endanger patients' lives.^7^ However, a close examination indicates that uncertainties and controversies still persist and await further research efforts to shed new light on them.

This meta‐analysis first followed a strict definition of “severity” and focused on the coagulation blood indicators, including platelet, d‐dimer, prothrombin time (PT), activated partial thromboplastin time (APTT), and fibrinogen. It attempted to explore the difference in coagulation dysfunction between severe and nonsevere adult COVID‐19 patients, so as to screen the severe patients and to timely adjust the therapeutic regimen to improve the prognosis.

## 2 METHODS

### 2.1 Data sources and search strategy

This meta‐analysis followed the PRISMA recommendations and was registered with PROSPERO—The International Prospective Register of Systematic Reviews (registration No. CRD42020186941). PubMed, Embase, Cochrane Central Register of Controlled Trials, and Web of Science were systematically searched for papers published between 1 December 2019 and 7 May 2020. The language restriction was English and the search terms were “COVID‐19” or “2019‐nCoV” or “SARS‐CoV‐2” or “Novel Coronavirus‐Infected Pneumonia” or “2019 novel coronavirus” or “coronavirus 2019” and “severe” or “severity.” The search strategy has been provided in the Supporting Information Material (File S1). The classification criteria for severity observed the “The American Thoracic Society (ATS) guidelines for community‐acquired pneumonia,”^8^ “WHO COVID‐19 Clinical Guidelines,”^9^ or “COVID‐19 Diagnosis and Treatment Protocol” of China.^10^ ATS guidelines for community‐acquired pneumonia,^8^ in which the severe type is defined according to either one major criterion (including septic shock in need of vasopressors and respiratory failure requiring mechanical ventilation) or three or more minor criteria (including respiration rate [RR] > 30/min, PaO_2_/FiO_2_ < 250 mm Hg, multilobar infiltrates, confusion/disorientation, uremia, leukopenia, thrombocytopenia, hypothermia, and hypotension requiring aggressive fluid resuscitation). WHO's interim guidelines for COVID‐19,^9^ in which the severe type includes severe pneumonia (for adolescents or adults: fever or suspected respiratory infection, plus one of the following: RR > 30/min; severe respiratory distress; or SpO_2_ ≤ 93% on room air), ARDS (PaO_2_/FiO_2_ ≤ 300mm Hg with positive end expiratory pressure or continuous positive airway pressure ≥5 cm H_2_O, or nonventilated; SpO_2_/FiO_2_ ≤ 315 or else), sepsis, and septic shock. The diagnostic and treatment guidelines for severe acute respiratory syndrome coronavirus 2 (SARS‐CoV‐2) issued by Chinese National Health Committee,^10^ in which the severe type is designated as one meeting any of the following indices: (a) respiratory distress with RR ≥ 30/min; (b) oxygen saturation at rest ≤93%; (c) PaO_2_/FiO_2_ ≤ 300 mm Hg; (d) respiratory failure requiring mechanical ventilation; (e) shock; and (f) requiring intensive care unit (ICU) admission requirement due to multiple organ failure.

### 2.2 Article eligibility criteria

After the removal of duplicates, two investigators (CHC and HC) independently evaluated study eligibility and inclusion by assessing the titles, abstracts, and the retrieved full texts. Disagreements were settled by consensus. The inclusion criteria were as follows: (a) confirmed cases of COVID‐19; (b) distinction of severe and nonsevere patients on the basis of clear and widely used criteria; (c) a minimum of 20 participants in the study. The exclusion criteria were as follows: (a) review articles, case reports, nonhuman studies and other forms; (b) scarce report of blood coagulation indicators; (c) studies of pediatric patients or pregnant women; (d) study subjects including patients less than 18 years old. Eligible studies were evaluated for the blood coagulation measurable in identifying the severity of the patients with COVID‐19.

### 2.3 Data extraction and quality assessment

Two reviewers (YH and HHM) independently extracted prespecified data elements (first author, year of publication, country, sample size, age, sex, value of coagulation indicators) from each study. Median and range were used to estimate mean and standard deviation (SD). The percentiles were converted to SD according to the following formula: SD ≈ Norm interquartile range = (P75 − P25) × 0.7413 (P75: 75th percentile and P25: 25th percentile).^11^ Study quality was assessed using the Newcastle‐Ottawa Scale (NOS) checklist. Disagreements were settled by consensus. A high‐quality study was defined as a study with a score ≥7.^12^

### 2.4 Data synthesis and analysis

All the statistical analyses were performed with the STATA software (version 12.0; STATA Corporation, College Station, TX). Weighted mean difference (WMD) with 95% confidence interval (CI) was calculated for each blood coagulation indicator. *I* ^2^ was employed to evaluate statistical heterogeneity with *I* ^2^ values of <25%, 25%‐75%, and >75%, respectively, indicating low, moderate, and high heterogeneity. The choice of the proper‐effect models was based on the analysis results: the fixed‐effect model was used for *I* ^2^ ≤ 50% and the random‐effect model for *I* ^2^ > 50%. The single‐arm meta‐analysis of proportions with 95% CIs was conducted for the value of blood coagulation indicators. Subgroup analysis was performed according to sample size, if adapting the random‐effect model. In addition, a further sensitivity analysis was performed to test the stability of the results. The Begg test was performed to assess publication bias if a coagulation indicator was retrieved from 10 or more studies. The statistical significance was set at *P* < .05.

## 3 RESULTS

### 3.1 Study selection

**Figure 1:**
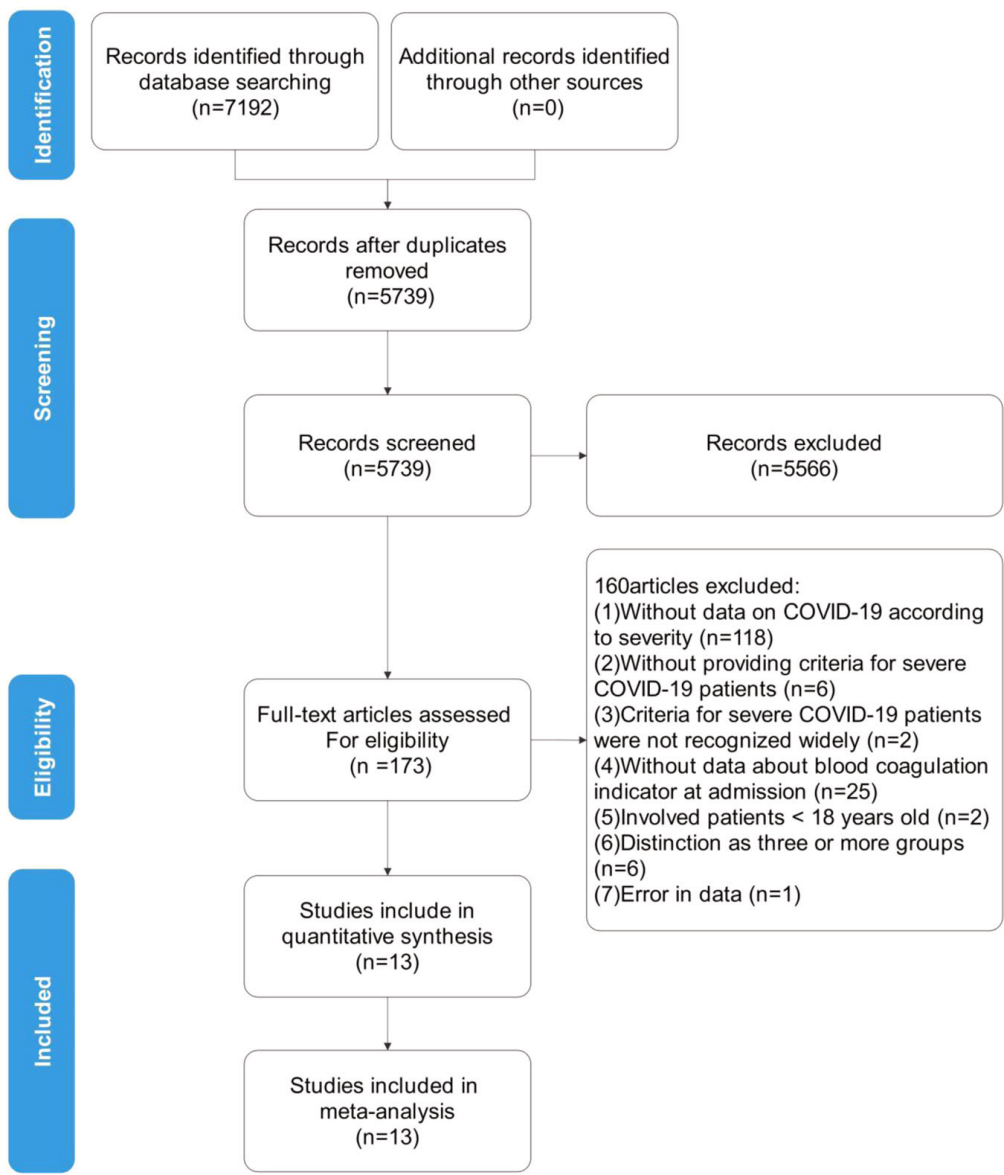
Flow‐chart of study selection

The review progress is summarized in Figure 1. A total of 7192 articles were identified basing on the search strategy, and 1453 duplications were removed. Then, 173 were potentially relevant to the review question after screening of titles and abstracts and 160 studies were further excluded according to the aforementioned criteria. Finally, 13 studies^13^, ^14^, ^15^, ^16^, ^17^, ^18^, ^19^, ^20^, ^21^, ^22^, ^23^, ^24^, ^25^ with 1341 patients and 371 severe COVID‐19 adults were enrolled in the meta‐analysis.

### 3.2 Study characteristics and quality assessment

All these studies were from China and published in 2020, with different sample sizes ranging from 21 to 204 patients, with a clear severity distinction (all the studies used the Chinese definition). The features of the 13 enrolled studies are summarized in Table 1. The quality assessment was based on NOS, with quality score ranging from 6 to 8. The quality results are shown in Table 1 and the assessment of each literature in the NOS scale is depicted in Table S1.

**Table 1:** Description of included studies

Study	Country	Year	Cases (severe/nonsevere)	Age, y (severe/nonsevere)	Sex (male [%]) (severe/nonsevere)	The value of blood coagulation indicator (severe/nonsevere)		The definition of “severity”	Quality score
Qu^13^	China	2020	3/27	60.0 (5.3)/49.4 (14.9)	NR	PLT	169.67 ± 48.95/192.26 ± 58.12	China	7
Gao^14^	China	2020	15/28	45.2 (7.7)/43.0 (14.0)	9 (60.0)/17 (60.7)	D‐D	0.49 (0.29‐0.91)/0.21 (0.19‐0.27)	China	8
						APTT	27.29 ± 6.09/30.42 ± 5.31		
						PT	11.26 ± 1.42/12.03 ± 1.21		
						FIB	3.84 ± 1.00/3.11 ± 0.83		
Wan^15^	China	2020	40/95	56 (52‐73)/44 (33‐49)	21 (52.5)/52 (54.7)	PLT	147 (118‐213)/170 (136‐234)	China	7
						D‐D	0.6 (0.4‐1.1)/0.3 (0.2‐0.5)		
						APTT	29.7 (22.62‐39.4)/26.6 (24.5‐28.8)		
Xie^16^	China	2020	28/51	62.5(50.5‐67.8)/59.0 (46.0‐66.0)	18 (64.3)/26 (51.0)	D‐D	0.7 (0.43‐2.36)/0.67 (0.31‐1.34)	China	6
Zheng^17^	China	2020	30/131	57 (46.5‐66.0)/40 (31‐51)	14 (46.7)/66 (50.4)	PLT	160 (133‐214)/171 (137‐221)	China	7
Zhang^18^	China	2020	58/82	64 (25‐87)/51.5 (26‐78)	33 (56.9)/38 (46.3)	D‐D	0.4 (0.2‐2.4)/0.2 (0.1‐0.3)	China	7
Chen^19^	China	2020	11/10	61.0 (56.5–66.0)/52.0 (42.8–56.0)	10 (90.9)/7 (70.0)	PLT	157 (134‐184.5)/175.6 (148.3‐194)	China	7
						D‐D	2.6 (0.6‐18.7)/0.3 (0.3‐0.4)		
						APTT	33.7 (32.1‐38.4)/44 (42.6‐47.6)		
						PT	14.3 (13.6‐14.6)/13.4 (12.8‐13.7)		
Liu^20^	China	2020	13/27	59.7 ± 10.1/43.2 ± 12.3	7 (53.8)/8 (29.6)	PLT	186.6 ± 68.1/181.4 ± 70.7	China	7
						D‐D	0.9 (0.7‐1.5)/0.4 (0.2‐0.8)		
						APTT	39.5 ± 4.2/39.5 ± 4.6		
						PT	13.4 ± 0.6/13.1 ± 0.6		
						FIB	6.3 ± 1.3/4.5 ± 1.4		
He^21^	China	2020	69/135	61 (52‐74)/43 (31‐53)	37 (53.62)/42 (31.11)	PLT	171 (138‐217)/200 (167‐261)	China	7
						D‐D	0.95 (0.41‐3.10)/0.32 (0.20‐0.70)		
						PT	11.8 (11.2‐12.7)/11.6 (11.0‐12.3)		
Chen^22^	China	2020	43/102	52.8 ± 15.5/45.3 ± 13.6	23 (53.5)/56 (54.9)	PLT	192 (142‐259)/204.5 (175‐254)	China	7
						D‐D	0.32 (0.21‐0.49)/0.24 (0.16‐0.39)		
						APTT	31.2 (28.5‐32.8)/29.2 (27.63‐31.85)		
						PT	11.9 (11.45‐12.5)/11.85 (11.3‐12.4)		
Zhu^23^	China	2020	16/111	57.50 ± 11.70/49.95 ± 15.52	9 (56.25)/73 (65.77)	PLT	155.00 (125.75‐206.00)/205.00 (165.00‐246.00)	China	7
						D‐D	0.16 (0.07‐0.28)/0.10 (0.08‐0.16)		
						FIB	5.74 (4.05‐6.68)/4.23 (3.63‐5.50)		
Fu^24^	China	2020	16/59	51.8 ± 12.8/45.1 ± 14.0	10 (62.5)/35 (59.32)	D‐D	0.32 (0.07‐1.22)/0.19 (0.07‐1.18)	China	7
						FIB	1.57 ± 0.39/0.94 ± 0.12		
Zheng^25^	China	2020	29/112	55 (47‐63)/45 (37‐55)	16 (55.2)/58 (51.7)	PLT	158 (125‐222)/203 (169‐246)	China	8

*Note:* China: The diagnostic and treatment guidelines for SARS‐CoV‐2 issued by Chinese National Health Committee. Data are mean ± SD, median (interquartile range).

Abbreviations: APTT, activated partial thromboplastin time; D‐D, d‐dimer; FIB, fibrinogen; NR, not reported; PLT, platelet; PT, prothrombin time

### 3.3 Meta‐analysis of coagulation indicators

**Figure 2:**
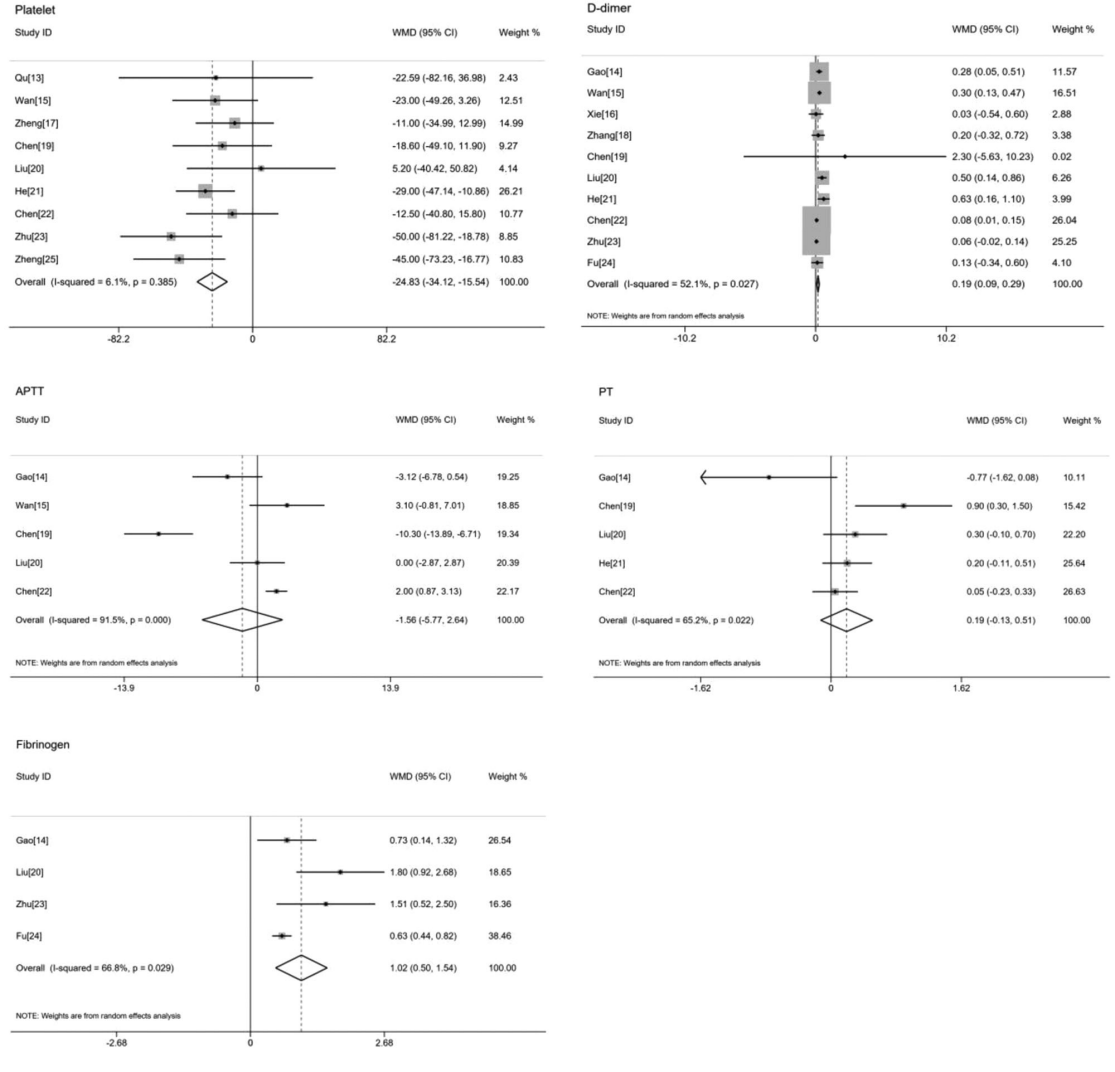
Forrest plots of the meta‐analyzed association of every blood coagulation indicator with the risk of severe 2019 novel coronavirus disease (COVID‐19). APTT, activated partial thromboplastin time; PT, prothrombin time; WMD, weighted mean difference

Five coagulation indicators (platelet, d‐dimer, APTT, PT, and fibrinogen) of the enrolled studies were analyzed and the results are shown in Figure 2.

#### 3.3.1 Platelet

**Figure 3:**
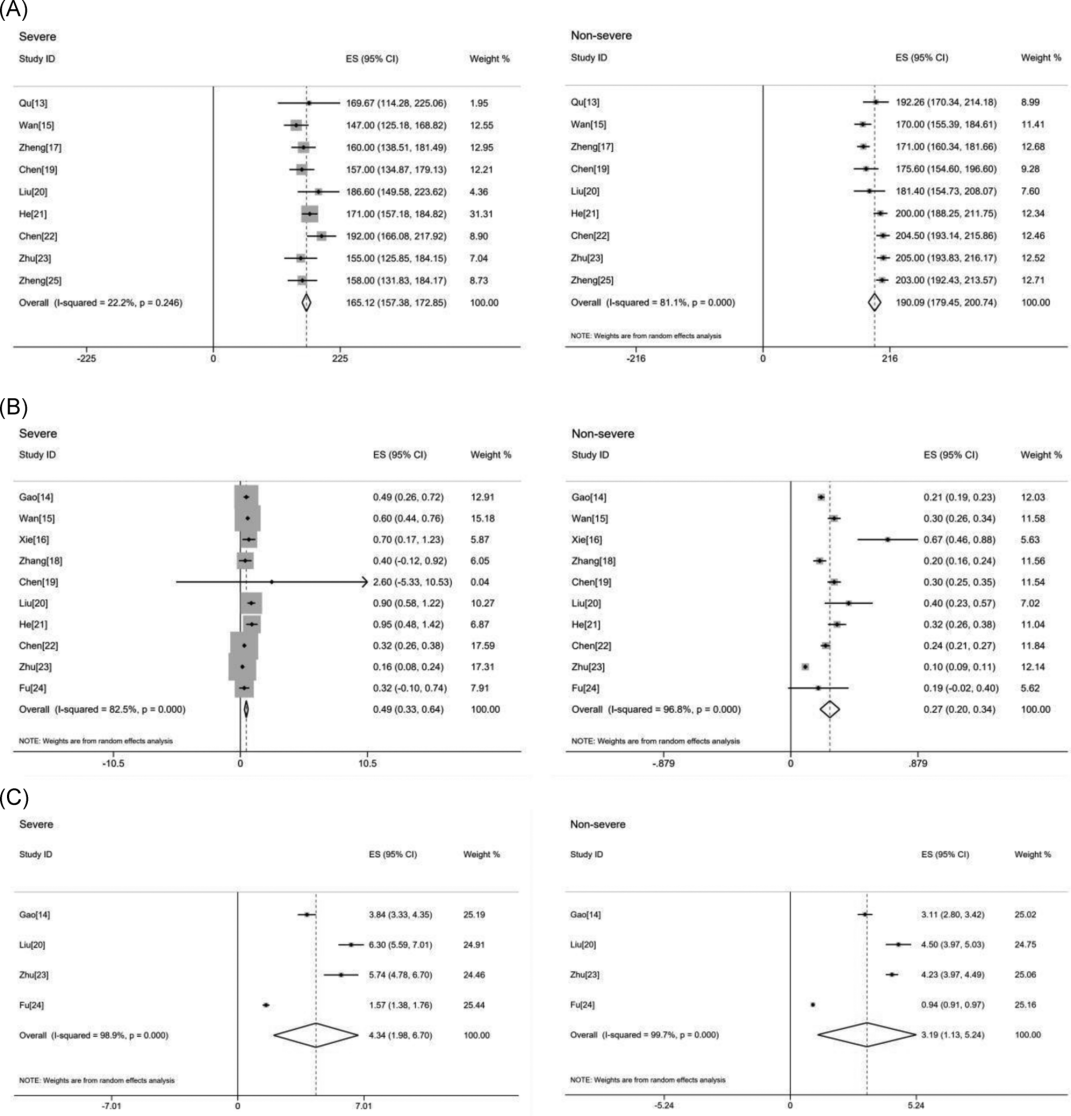
(A) Platelet; (B) d‐dimer; (C) fibrinogen. Forest plot of the value of blood coagulation indicators in severe and nonsevere COVID‐19 patients, respectively. COVID‐19, 2019 novel coronavirus disease; ES, effect size

Nine studies with 750 nonsevere and 385 severe COVID‐19 patients were eligible for the meta‐analysis. The fixed‐effect model demonstrated that the severe group had a markedly lower platelet than the nonsevere group (WMD = −24.83, 95% CI: −34.12 to −15.54; *P* < .001) without evident heterogeneity (*I* ^2^ = 6.1%; Figure 2). According to the single‐arm meta‐analysis, the pooled platelet in the severe group was 165.12 (95% CI: 157.38‐172.85) and that of the nonsevere group was 190.09 (95% CI: 179.45‐200.74; Figure 3).

#### 3.3.2 D‐dimer

Ten studies assessed coagulation function using d‐dimer in comparing 309 severe and 700 nonsevere COVID‐19 patients. The value of d‐dimer was higher in the severe group when compared with the nonsevere group (WMD = 0.19, 95% CI: 0.09‐0.29; *P *< .001), with a moderate heterogeneity (*I* ^2^ = 52.1%) in the random‐effect model (Figure 2). Results of the single‐arm meta‐analysis showed that for severe patients, the pooled d‐dimer was 0.49 (95% CI: 0.33‐0.64) and that of the nonsevere group was 0.27 (95% CI: 0.20‐0.34; Figure 3).

#### 3.3.3 APTT

Five studies analyzed APTT, involving 262 nonsevere and 122 severe COVID‐19 patients. The pooled WMD was −1.56 (95% CI: −5.77 to 2.64; *P* = .465) with a substantial heterogeneity (*I* ^2^ = 91.5%; Figure 2). Despite the high heterogeneity, the result should be interpreted with caution, for the 95% CI of WMD ranged from −5.77 to 2.64.

#### 3.3.4 PT

For PT, five studies with 302 nonsevere and 151 severe COVID‐19 patients were eligible for meta‐analysis. In four studies, PT was higher in the severe group than in the nonsevere one, but the difference was not significant (WMD = 0.19, 95% CI: −0.13 to 0.51; *P* = .243, *I* ^2^ = 65.2%; Figure 2).

#### 3.3.5 Fibrinogen

A total of four studies with 225 nonsevere and 60 severe COVID‐19 patients were included in the meta‐analysis. The analysis of the random‐effect model showed that compared with the nonsevere group, the severe group had a higher fibrinogen (WMD = 1.02, 95% CI: 0.50‐1.54; *P* < .001) with a moderate heterogeneity (*I* ^2^ = 66.8%; Figure 2). The single‐arm meta‐analysis showed that the pooled fibrinogen was 4.34 (95% CI: 1.98‐6.7) in the severe group but 3.19 (95% CI: 1.13‐5.24) in the nonsevere group (Figure 3).

### 3.4 Subgroup analysis

**Figure 4:**
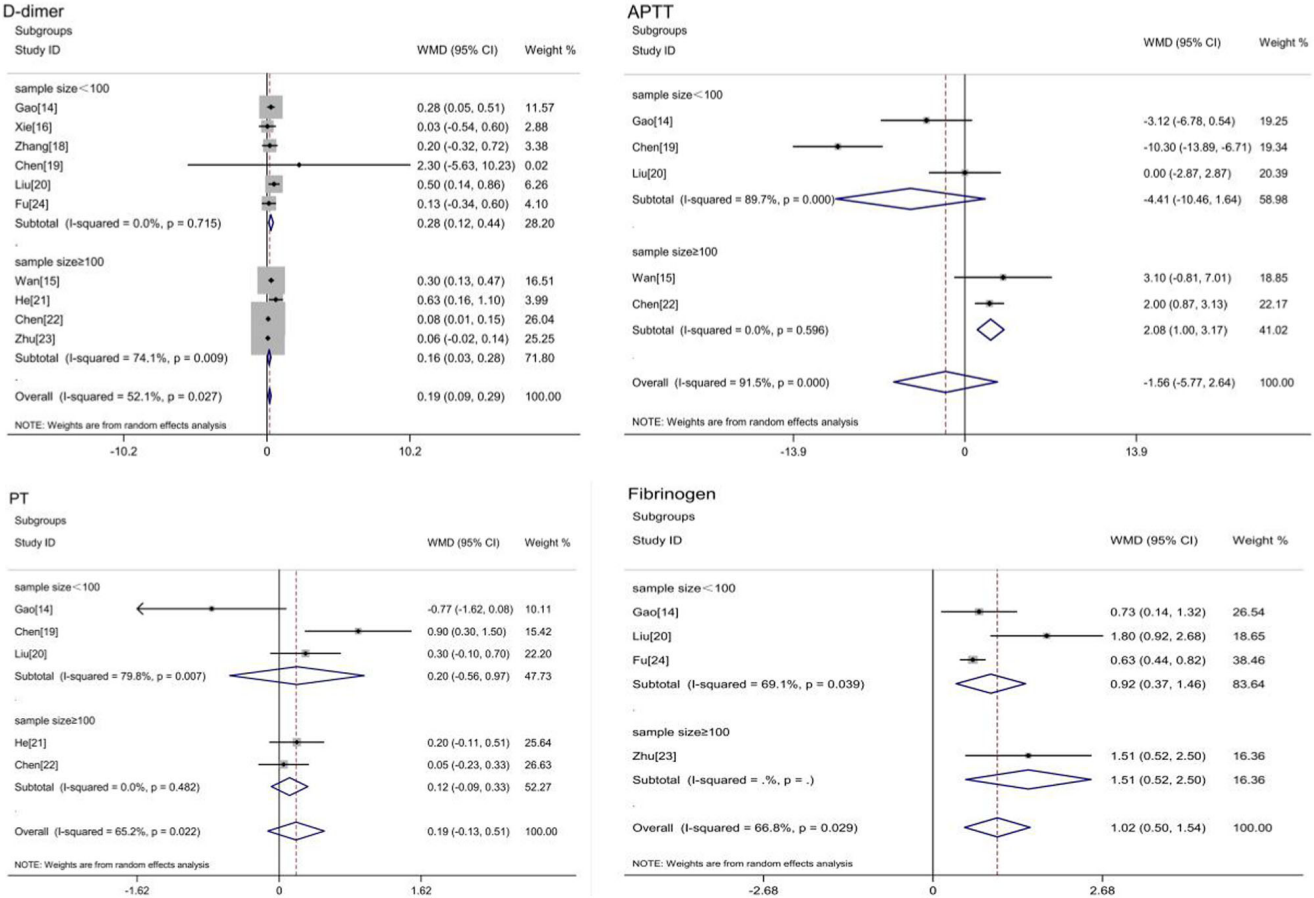
Subgroup analysis according to sample size of coagulation indicators in severe or nonsevere COVID‐19. COVID‐19, 2019 novel coronavirus disease

Subgroup analysis based on the sample size showed that there was an association between the d‐dimer levels and the severity of COVID‐19 in both sample size <100 subgroup and sample size ≥100 subgroup (sample size <100: WMD = 0.28, 95% CI: 0.12‐0.44; *I* ^2^ = 0%; sample size ≥100: WMD = 0.16, 95% CI: 0.03‐0.28; *I* ^2^ = 74.1%; Figure 4). APTT increased in the severe COVID‐19 in the subgroup with sample size ≥100 (WMD = 2.08, 95% CI: 1.00‐3.17; *I* ^2^ = 0%; Figure 4). There was no association between the PT levels and the severity of COVID‐19 in two subgroups (Figure 4). Fibrinogen increased in the severe COVID‐19 despite the subgroup based on sample size (Figure 4).

### 3.5 Sensitivity analysis and publication bias

**Figure 5:**
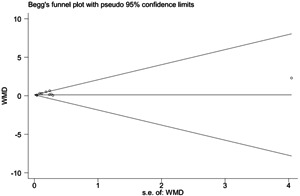
Begg's funnel plot for d‐dimer in comparing severe and nonsevere COVID‐19

As shown in Figure S1, from the results of the sensitivity analysis, the combined results did not change with the exclusion of any of the studies. Thus, sensitivity analysis suggested that these meta‐analyses (d‐dimer, APTT, PT, and fibrinogen) were steady. Because only d‐dimer was retrieved from 10 studies (≥10), a funnel plot regarding the d‐dimer showed that the *P‐*value of the Begg test was .858. The Begg test of “d‐dimer” suggested that no stable evidence of publication bias was present in the meta‐analysis (Figure 5).

## 4 DISCUSSION

To assess the differences in coagulation dysfunction between severe and nonsevere adult COVID‐19 patients, the current meta‐analysis adopted a well‐established definition of disease severity and systematically evaluated five coagulation indicators (platelet, d‐dimer, APTT, PT, and fibrinogen). The results showed that significant differences in platelet, d‐dimer, and fibrinogen were evident between severe and nonsevere patients though without significant differences in PT and APTT. The findings suggest that lower platelet, higher d‐dimer and fibrinogen are risk factors for increased severity of COVID‐19.

Previous studies suggest that influenza‐associated pneumonia is associated with thrombotic events.^26^ Similarly, COVID‐19 can induce thromboembolic complications, especially in severe patients.^27^ In severe cases, COVID‐19 triggers a cytokine storm, which activates the coagulation cascade, resulting in the thrombotic phenomena.^28^ Nevertheless, the mechanism of COVID‐19‐related coagulation disorder is far more complicated to be explained by inflammation.^29^, ^30^ A previous study proposed that the immunity‐related pulmonary intravascular coagulation disease differs from sepsis‐induced coagulation dysfunction and DIC, with the latter characterized by platelet reduction and PT prolongation.^31^ So COVID‐19‐related coagulation dysfunction may have its own characteristics.

Thrombocytopenia is considered as a dysregulated host response in critical sepsis patients,^32^ which usually occurs after a viral infection (such as influenza and HIV).^33^ In SARS‐related diseases, the virus has been suspected to cause platelet consumption in the lungs by damaging epithelial cells^34^ and infect hematopoietic stem cells and megakaryocytes.^35^ Similarly, COVID‐19 is homologous to SARS. A recent study by Yang et al^36^ showed that 20.7% of patients with COVID‐19 had a low platelet (<125 × 10^9^/L) and 5% of them had a very low platelet (range, 0‐50 × 10^9^/L) with 92.1% mortality.^36^ However, the incidence of thrombocytopenia was much lower in a study by Zheng et al^37^ (8.5% vs 20.7%), which may be explained by the fact that the study enrolled fewer severe cases. Still strangely, a study by Panigada et al^38^ found that platelet was normal or increased by thromboelastography in a small lCU COVID‐19 sample. Two meta‐analysis studies^39^, ^40^ also touched upon platelet in screening severe patients—one found no significant difference in platelet between the severe and mild patients, with only five studies and many severity criteria^39^; the other reported that thrombocytopenia was associated with the severity of COVID‐19, but with a high heterogeneity (*I* ^2^ = 92%).^40^ Compared with the two meta‐analysis studies, the current meta‐analysis has a clearer definition of “severity,” focuses on “adult patients,” and enrolls more studies. Therefore, the heterogeneity is low and the results are more accurate.

COVID‐19 is associated with thromboembolism events. Studies have shown that 30% of patients with COVID‐19 were complicated by pulmonary thromboembolism^41^ and that the incidence of venous thromboembolism (VTE) was 47% in patients who were admitted to ICU after 14 days.^42^ Pulmonary microvascular dysfunction may be an important cause of hypoxemia in patients with COVID‐19. Meanwhile, autopsy revealed that pulmonary embolism was the direct cause of death in 4 of 12 patients with COVID‐19.^43^ Hence, thrombogenesis is significant in COVID‐19 cases, given that d‐dimer plays a key role in thromboembolism events. Our meta‐analysis is consistent with the listed findings: d‐dimer was elevated in the severe COVID‐19 patients. Elevated d‐dimer reflects hypercoagulable state and VTE events, which is associated with ARDs and death of COVID‐19 patients.^44^ Long‐term bedridden condition, obesity, smoking, and advanced age not only are the risk factors for COVID‐19 severity but also lead to increased d‐dimer.^45^, ^46^ In addition, liver involvement in the severity of COVID‐19 cases^47^ may lead to insufficient synthesis of coagulation factors, resulting in hyperplasminolysis and increased d‐dimer.^48^

Fibrinogen is an essential part of the blood coagulation cascade. In sepsis, decreased fibrinogen is associated with an increased mortality,^49^ which is related to consumptive coagulopathy. The elevated fibrinogen has been documented in infectious diseases, acute stroke, and myocardial infarction.^50^ The current study is the first meta‐analysis to summarize increased fibrinogen levels in severe COVID‐19 adults. In addition, fibrinogen is an acute‐phase protein, which is induced by interleukin 6 and associated with inflammatory responses.^51^ Once infection occurs, hepatic synthesis of fibrinogen increases 2 to 10 times.^52^ Possibly different from septic coagulation disorder, early severe COVID‐19 patients present hypercoagulability rather than consumptive coagulopathy.^53^, ^54^

Prolonged PT and APTT are linked to anticoagulant, coagulation factor deficiency, and fibrinolysis, which have been used as laboratory tools to predict bleeding.^55^, ^56^ The performance of PT and APTT was contradictory in the initial research of severe COVID‐19 cases. Some studies showed shortened PT and APTT in severe COVID‐19 patients^14^ while another study reported prolonged PT and APTT.^22^ Our meta‐analysis found no difference in PT and APTT between the severe and nonsevere groups upon admission. Probably, PT and APTT may fail in an early recognition and be influenced by many factors (eg, anticoagulant). However, this finding should be interpreted with caution because of the high heterogeneity and much less included literature. More clinical trials are urgently needed to investigate the relation between PT/APTT and COVID‐19 severity.

## 5 LIMITATIONS

The following limitations should be mentioned: first, patients from the studies were all from China. There may exist racial differences in blood coagulation.^57^ Further studies on coagulation from other races are highly awaited. Second, blood indicators were included upon admission, without considering previous use of antiplatelet or anticoagulant drugs. Finally, not all five indicators were included in some studies, so we hope that more studies related to blood coagulation will expand the sample size in the future.

## 6 CONCLUSIONS

Coagulation dysfunction is closely related to the severity of COVID‐19 cases and affects the prognosis of COVID‐19 patients. Low platelet, high d‐dimer and fibrinogen may serve as risk indicators for the progression of COVID‐19 severity in the early screening of severe and nonsevere COVID‐19 patients. Further exploration of coagulation function is crucial for prophylactic and anticoagulation therapy in severe COVID‐19 cases.

## AUTHOR CONTRIBUTIONS

JL and HY designed the study and drafted and revised the manuscript. HC, CH, HH, and SZ acquired the data. CL organized the figures. KL and SS contributed equally to this study, provided supervision, and critically revised the manuscript. The authors approved the final version of the manuscript and agreed to be accountable for all aspects of the study.

## Supporting information

Supplementary information

Supplementary information

Supplementary information

## Data Availability

All data are fully available online without restriction.
