# Supplementary material for: COVID‐19 and coagulation dysfunction in adults: A systematic review and meta‐analysis
Source: J Med Virol. 2020 Aug 2;93(2):934–44. doi: 10.1002/jmv.26346 (PMC7405098; doi:10.1002/jmv.26346)
Supplement: Supplementary file 1 — Supplementary information [file JMV-93-934-s001.docx]

**Supplementary file 1: the search strategy and search results**

**Pubmed**

**Search strategy:**

((((("COVID-19") OR ("Novel Coronavirus–Infected Pneumonia")) OR ("2019 novel coronavirus")) OR ("2019-nCoV")) OR ("SARS-CoV-2")) OR ("coronavirus 2019") AND (("severe") OR ("severity")) [All Fields]

**Search results:** 4296

**Cochrane**

**Search strategy:**

#1 "COVID-19"

#2 "Novel Coronavirus–Infected Pneumonia"

#3 "2019 novel coronavirus"

#4 "2019-nCoV"

#5 "SARS-CoV-2"

#6 "coronavirus 2019"

#7 #1 or #2 or #3 or #4 or #5 or #6

#8 "severe" or "severity"

#9 #7 and #8 [All Text]

**Search results:** 165

**Web of science**

**Search strategy:**

#1 "COVID-19"

#2 "Novel Coronavirus–Infected Pneumonia"

#3 "2019 novel coronavirus"

#4 "2019-nCoV"

#5 "SARS-CoV-2"

#6 "coronavirus 2019"

#7 #1 or #2 or #3 or #4 or #5 or #6

#8 "severe" or "severity"

#9 #7 and #8 [Topic]

**Search results:** 585

**Embase**

**Search strategy:**

#1 "COVID-19"

#2 "Novel Coronavirus–Infected Pneumonia"

#3 "2019 novel coronavirus"

#4 "2019-nCoV"

#5 "SARS-CoV-2"

#6 "coronavirus 2019"

#7 #1 or #2 or #3 or #4 or #5 or #6

#8 "severe" or "severity"

#9 #7 and #8 [All Fields]

**Search results:** 2146
