# Supplementary material for: COVID‐19 and coagulation dysfunction in adults: A systematic review and meta‐analysis
Source: J Med Virol. 2020 Aug 2;93(2):934–44. doi: 10.1002/jmv.26346 (PMC7405098; doi:10.1002/jmv.26346)
Supplement: Supplementary file 2 — Supplementary information [file JMV-93-934-s002.docx]

| Table S1 | | | | | | | | | | | |
| --- | --- | --- | --- | --- | --- | --- | --- | --- | --- | --- | --- |
| Quality assessments of studies based on Newcastle Ottawa Scale | | | | | | | | | | | |
| Author | Years | Selection | | | | Comparability | | Exposure | | | Total score |
|  |  | a | b | c | d | e | f | g | h | i |  |
| Qu^[13]^ | 2020 | * | * | - | * | * | * | * | * | - | 7 |
| Gao^[14]^ | 2020 | * | * | - | * | * | * | * | * | * | 8 |
| Wan^[15]^ | 2020 | * | * | - | * | * | * | * | * | - | 7 |
| Xie^[16]^ | 2020 | * | - | - | * | * | * | * | * | - | 6 |
| Zheng^[17]^ | 2020 | * | * | - | * | * | * | * | * | - | 7 |
| Zhang^[18]^ | 2020 | * | * | - | * | * | * | * | * | - | 7 |
| Chen^[19]^ | 2020 | * | * | - | * | * | * | * | * | - | 7 |
| Liu^[20]^ | 2020 | * | * | - | * | * | * | * | * | - | 7 |
| He^[21]^ | 2020 | * | * | - | * | * | * | * | * | - | 7 |
| Chen^[22]^ | 2020 | * | * | - | * | * | * | * | * | - | 7 |
| Zhu^[23]^ | 2020 | * | * | - | * | * | * | * | * | - | 7 |
| Fu^[24]^ | 2020 | * | * | - | * | * | * | * | * | - | 7 |
| Zheng^[25]^ | 2020 | * | * | - | * | * | * | * | * | * | 8 |
| Qulity assessment checklist: a: Is the case definition adequate? b: Representativeness of the Cases. | | | | | | | | | | | |
| c: Selection of Controls. d: Definition of Controls. e: Study controls for ethnicity. | | | | | | | | | | | |
| f: Study controls for any additional factor. g: Ascertainment of exposure. | | | | | | | | | | | |
| h: Same method of ascertainment for cases and controls. i: Non-response rate. | | | | | | | | | | | |
