# Supplementary figures and images for: COVID‐19 and coagulation dysfunction in adults: A systematic review and meta‐analysis
Source: J Med Virol. 2020 Aug 2;93(2):934–44. doi: 10.1002/jmv.26346 (PMC7405098; doi:10.1002/jmv.26346)

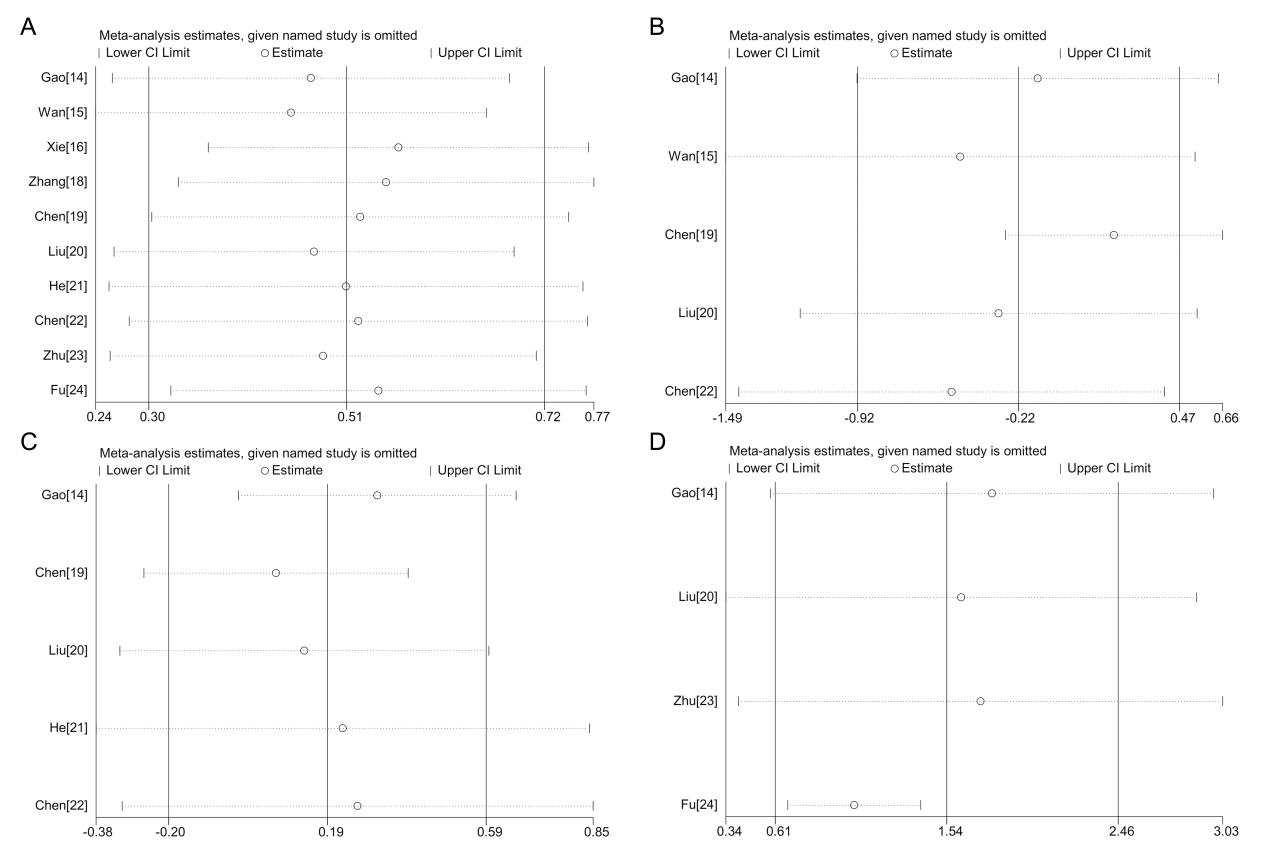


Figure S1. Sensitivity analyses by excluding one study at a time. (A)D-dimer; (B)APTT; (C)PT;(D)Fibrinogen.

Supplement: Supplementary file 3 — Supplementary information [file JMV-93-934-s003.docx]
